# Fibroblast Growth Factor Receptor 4 Promotes Triple‐Negative Breast Cancer Progression via Regulating Fatty Acid Metabolism Through the AKT/RYR2 Signaling

**DOI:** 10.1002/cam4.70439

**Published:** 2024-12-10

**Authors:** Jinhui Ye, Song Wu, Qiang Quan, Feng Ye, Jinhui Zhang, Cailu Song, Yidan Fan, Huijiao Cao, Hailin Tang, Jianfu Zhao

**Affiliations:** ^1^ Research Center of Cancer Diagnosis and Therapy, Department of Oncology The First Affiliated Hospital of Jinan University Guangzhou China; ^2^ Department of Breast Oncology The First People's Hospital of Zhaoqing Zhaoqing China; ^3^ State Key Laboratory of Oncology in South China, Guangdong Provincial Clinical Research Center for Cancer Sun Yat‐Sen University Cancer Center Guangzhou China

**Keywords:** AKT, fatty acid metabolism, FGFR4, RyR2, TNBC

## Abstract

**Background:**

Triple‐negative breast cancer (TNBC) is the most aggressive subtype of breast cancer. Previous studies have found that fibroblast growth factor receptor 4 (FGFR4) plays a crucial role in tumor development and metastasis. However, the potential role and underlying mechanisms of FGFR4 in the progression of TNBC remain unclear.

**Methods:**

Statistical analysis of FGFR4 expression data in public databases was used to reveal its role in TNBC. qRT‐PCR was used to detect FGFR4 expression levels. The impact of FGFR4 level changes on TNBC cell proliferation was assessed using CCK‐8 and colony formation assays, while Transwell invasion assays and JC‐1 staining were employed to analyze the effects of FGFR4 level changes on the invasiveness and survival capability of TNBC cells. Differentially expressed genes were subjected to Gene Ontology, KEGG, and GSEA enrichment analyses to identify associated signaling pathways. Additionally, Oil Red O staining, fatty acid metabolite detection, and Western blot analysis were used to investigate the impact of FGFR4 and its inhibitor fisogatinib, as well as the AKT activator SC79, on the metabolic reprogramming of fatty acids in TNBC cells.

**Results:**

FGFR4 was found to be upregulated in breast cancer and correlated with poorer patient outcomes. Inhibition of FGFR4 resulted in reduced cell growth and invasion in TNBC cells. It also led to increased lipid accumulation, upregulated lipid biosynthesis‐related genes, and downregulated lipolysis‐related genes. Mechanistically, FGFR4 inhibition suppressed the activation of the AKT/RYR2 signaling pathway. Reactivating the AKT pathway reversed the suppressive effects of FGFR4 inhibition on TNBC progression.

**Conclusion:**

Dysregulated FGFR4 activates the AKT/RYR2 axis, leading to tumor proliferation, invasion, and altered lipid metabolism in TNBC. FGFR4 inhibition could potentially serve as a novel therapeutic strategy for TNBC treatment.

**Abbreviations**
ACC: acetyl‐CoA carboxylase
ATGL: adipose triglyceride lipase
FABP4: fatty acid‐binding protein 4
FASN: fatty acid synthase
FFA: free fatty acid
FGFR4: fibroblast growth factor receptor 4
HSL: hormone‐sensitive lipase
MMP: mitochondrial membrane potential
RyR2: ryanodine receptor 2
TG: triglyceride
TNBC: triple‐negative breast cancer

## 1 Introduction

Globally, breast cancer is the predominant form of cancer diagnosed in women and serves as the principal cause of cancer‐related mortality among this demographic [1]. Although the comprehensive treatment for breast cancer has greatly improved the treatment effect of breast cancer, the prognosis of triple‐negative breast cancer (TNBC) has not been fully and effectively controlled, which still shows a high mortality rate [2, 3, 4]. Deeply studying the mechanism of tumor progression of TNBC and developing new treatment methods are the breakthrough to improve the curative effect and the prognosis of TNBC [5, 6, 7, 8, 9].

Metabolic reprogramming is a hallmark of cancer, and cancer cells could change metabolism to promote proliferation and survival. Recently, lipid metabolism is getting more and more attention in cancer research. In TNBC, lipid deregulation widely exists and could lead to subsequent cancer progression, such as cell proliferation, metastasis, and chemoresistance. For example, circMyc was found to elevate in TNBC and promoted TNBC proliferation and invasion by modulating metabolic reprogramming and inducing lipogenesis [10]. Thus, identifying metabolic‐related targets and developing lipid‐based treatment could provide promising starting points against TNBC [11].

The fibroblast growth factor receptor (FGFR) family, classified as a tyrosine kinase receptor family, is instrumental in mediating a multitude of biological processes, most notably cell proliferation [12]. Fibroblast growth factor receptor 4 (FGFR4), as a member of the FGFR family, is involved in tumorigenesis and tumor progress in various tumors [13]. Elevated expression of FGFR4 in cancers could result in enhanced cell proliferation and tumor growth [14]. There are only a few researches about FGFR4 in breast cancer so far. In TNBC, FGFR4 was identified as a key driver of cell growth via activating the PI3K/AKT signaling [15]. Overexpression of FGFR4 was associated with worse outcome of breast cancer patients. And FGFR4 promoted cell proliferation and suppressed cell apoptosis via activating MST1/2 in breast cancer [16]. In spite of the above findings, the possible roles and underlying mechanisms of FGFR4 in TNBC lipid metabolism have not yet been explored.

Here in this study, we found that FGFR4 was upregulated in breast cancer and correlated with poorer outcome. Subsequent experiments revealed that inhibition of FGFR4 led to repressed cell growth and invasion in TNBC. Moreover, FGFR4 could regulate fatty acid metabolism in TNBC cells. Mechanically, we revealed that dysregulation of FGFR4 led to activation of the AKT/RYR2 axis and the following tumor proliferation, invasion, and lipid metabolism reprogramming in TNBC. The PI3K/AKT axis dysregulation frequently happens in TNBC [17]. And dysregulation of ryanodine receptor 2 (RyR2) is also related to cancer development and progression in TNBC [18]. Inhibition of FGFR4 could potentially function as a novel therapeutic strategy for TNBC treatment in the future.

## 2 Methods

### 2.1 Collection and Analysis of Data

The TCGA‐BRCA cohort, the pan‐BRCA cohorts from bc‐GenExMiner (https://bcgenex.ico.unicancer.fr/BC‐GEM/) and BEST databases (https://rookieutopia.hiplot.com.cn/app_direct/BEST/), and pan‐cancer cohorts (TCGA + GTEx) (https://www.xiantaozi.com/) were collected for FGFR4 expression, prognosis, and functional analysis. For survival analysis, the FGFR4 “high” and “low” populations were defined using the “best cut‐off” method using “survminer” package.

### 2.2 Tissue Specimens

During surgery at Sun Yat‐Sen University Cancer Center Hospital, 30 paired TNBC samples (Tumor) and adjacent normal samples (Normal) were collected. RNAlater (Invitrogen, USA) was used to preserve fresh tissues for quantitative real‐time polymerase chain reaction (qRT‐PCR) assays. Status of ER, PR, and HER‐2 were confirmed by IHC in the Pathology Department of Sun Yat‐Sen University Cancer Center. This study was approved by Sun Yat‐Sen University Cancer Center, and 1964 Helsinki Declaration was followed. Patients involved had written informed consent forms.

### 2.3 Cell Lines and Culture

Human normal breast cell line (MCF‐10A) and human breast cancer cell lines (HER‐2 positive: SKBR3 and BT474; Luminal A/B: MCF7 and T47D; TNBC: MDA‐MB‐231, BT549, and SUM159) were bought from ATCC (USA) and cultured with RPMI‐1640 at 37°C with 5% CO_2_.

### 2.4 Quantitative Real‐Time Polymerase Chain Reaction

RNAs were isolated with TRIzol (Invitrogen, USA). mRNA expression was detected with SYBR Premix Ex Taq (Takara, China) with Roche LightCycler 480 II. The primers were bought from Invitrogen: FGFR4 forward, 5′‐CCATAGGGACCCCTCGAATAG‐3′, reverse, 5′‐CAGCGGAACTTGACGGTGT‐3′; GAPDH forward, 5′‐ACAACTTTGGTATCGTGGAAGG‐3′, reverse, 5′‐GCCATCACGCCACAGTTTC‐3′. The relative mRNA levels were calculated by 2^−△△*CT*^.

### 2.5 CCK‐8 Assay

MDA‐MB‐231 and BT549 cells were seeded in 96‐well plates (3 × 10^3^ cells/well) and treated with Fisogatinib (FGFR4‐specific inhibitor, also known as BLU‐554, Selleck, #S8503, USA, 100 nmol/L). Twenty‐four hours later, cells were treated with CCK‐8 reagent (Beyotime, China) and cultured at 37°C for 1 h. Finally, the absorbance at 450 nm was detected and analyzed.

### 2.6 Colony Formation Assay

MDA‐MB‐231 and BT549 cells were seeded in six‐well plates (1 × 10^3^ cells/well) and treated with Fisogatinib (100 nmol/L cell) with or without SC79 (AKT activator, MCE, #HY‐18749, USA, 4 μg/mL). Two weeks later, 4% paraformaldehyde was used to fix the colonies and 0.5% crystal violet was used to stain the colonies. Finally, the colony numbers were counted.

### 2.7 Transwell Assay

Briefly, MDA‐MB‐231 and BT549 cells (3 × 10^4^ cells/well) were treated with Fisogatinib (100 nmol/L cell) with or without SC79 (4 μg/mL) and then re‐suspended and introduced to the upper chambers (FBS‐free medium), and 20% of FBS medium was introduced to the lower chambers (BD Biosciences). The upper compartment's Matrigel was removed 48 h later, while the infiltrating cells adherent to the bottom were fixed with Methanol. The invasive cells were photographed and enumerated after being stained with crystal violet (2%).

### 2.8 Mitochondrial Membrane Potential Detection

MMP was detected with Mitochondrial Membrane Potential (MMP) Assay Kit (Elabscience, China). MDA‐MB‐231 and BT549 cells (5 × 10^5^ cells/well) were cultured in 24‐well plates treated with Fisogatinib (100 nmol/L) with or without SC79 (4 μg/mL). Then, cells were washed with 1 × JC‐1 Assay Buffer and incubated with JC‐1 for 20 min at 37°C. Then, cells were washed with 1 × JC‐1 Assay Buffer and observed under a confocal microscope.

### 2.9 Oil Red O Staining

Briefly, MDA‐MB‐231 and BT549 cells were seeded and then treated with Fisogatinib (100 nmol/L cell) with or without SC79 (4 μg/mL). After fixing by 4% paraformaldehyde, cells were stained by Oil red O reagent (Beyotime) to detect the lipid droplet (LD) levels. At last, the cells were photographed under a light microscope.

### 2.10 Detection of Fatty Acid Metabolism

The levels of triglyceride (TG), phospholipid, and free fatty acid (FFA) of MDA‐MB‐231 and BT549 cells were detected with Triglyceride Assay Kit (Abcam, USA), Phospholipid Assay Kit (Abcam), and Free Fatty Acid Quantification Kit (Abcam) according to the instructions of the manuals.

### 2.11 Western Blotting

Protein was isolated with RIPA lysis buffer and PMSF, and the protein was separated via 10% SDS‐PAGE. Then, the protein was transferred onto PVDF membranes and blocked with 5% skim milk for an hour. After that, PVDF membranes were incubated with antibodies including FGFR4 (1:1000, #AF7762, Affinity, USA), fatty acid synthase (FASN, 1:1000, #DF6106, Affinity), fatty acid‐binding protein 4 (FABP4, 1:500, #DF6035, Affinity), acetyl‐CoA carboxylase (ACC, 1:500, #AF6421, Affinity), hormone‐sensitive lipase (HSL, 1:500, #AF6403, Affinity), adipose triglyceride lipase (ATGL, 1:1000, #DF7756, Affinity), AKT (1:1000, #AF6261, Affinity), p‐AKT(Ser473) (1:1000, #AF0016, Affinity), RYR2 (1:1000, #AF0015, Affinity), p‐RYR2(Ser2808) (1:1000, #AF7454, Affinity) and GAPDH (1:10000, #AF7021, Affinity) at 4°C. The next day, the PVDF membranes were incubated with secondary antibodies (1:5000, #S0001, Affinity) for an hour at room temperature. At last, protein bands were detected with enhanced chemiluminescence reagent (Yeasen), and relative grayscale was quantified by ImageJ software.

### 2.12 Statistical Analysis

Statistical analysis was conducted with GraphPad Prism 9.0. Significance was confirmed when *p* ≤ 0.05 by Student's *t* test and ANOVA test. All the assays were conducted thrice and displayed with mean ± SD of three repeated experiments.

## 3 Results

### 3.1 FGFR4 Is Upregulated and Correlated With Worse Outcome of Breast Cancer

**FIGURE 1:**
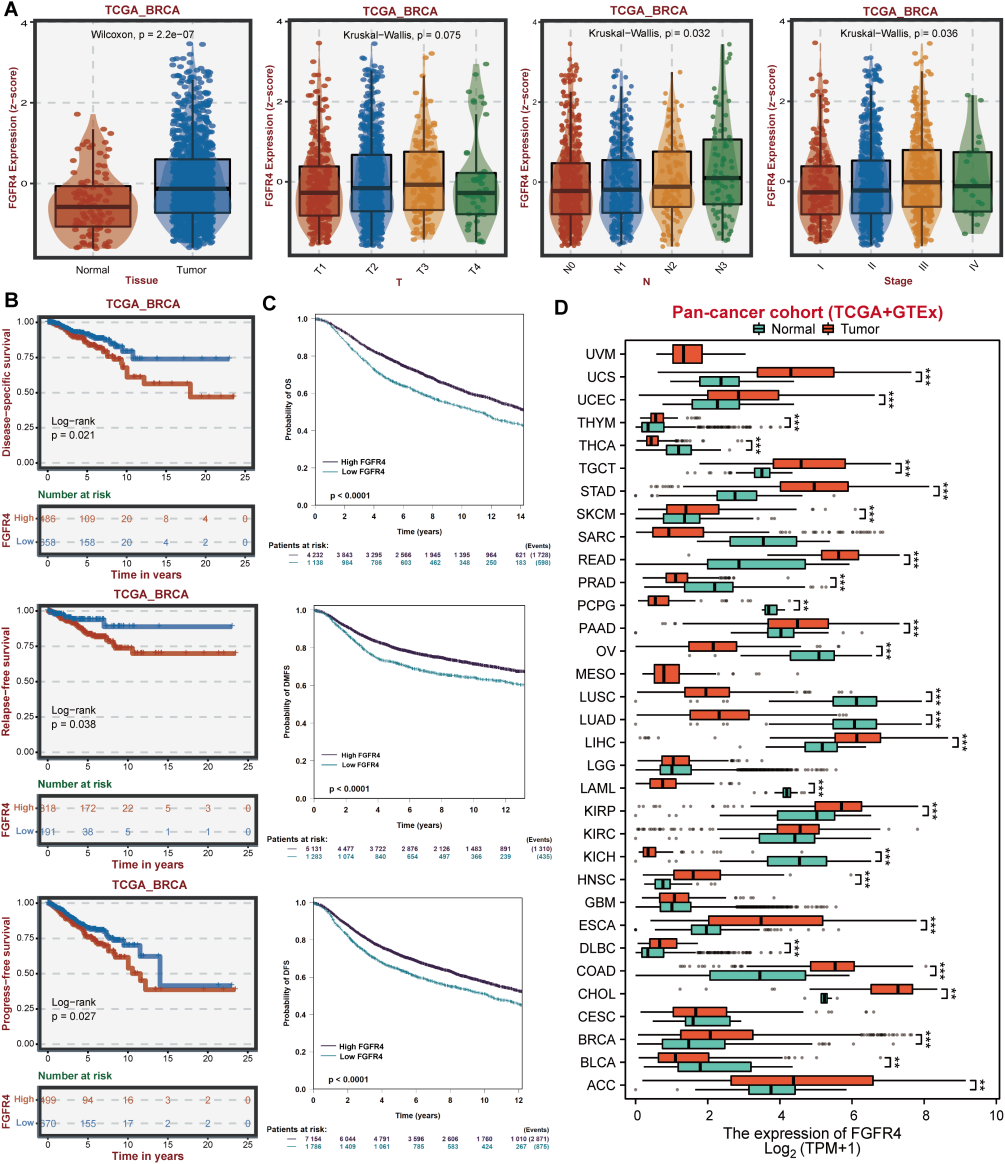
FGFR4 is upregulated and correlated with worse outcome of breast cancer. (A) Boxplots of the relationship between the expression of FGFR4 and tissue type (Normal or Tumor), tumor size (T1, T2, T3, and T4), lymph node metastatic status (N0, N1, N2, and N3), and tumor stage (I, II, III, and IV) in the TCGA‐BRCA cohort. (B) K‐M analyses of the prognostic value of FGFR4 in the TCGA‐BRCA cohort. (C) K‐M analyses of the prognostic value of FGFR4 in the pan‐BRCA cohorts from bc‐GenExMiner database. (D) Boxplots of the differences of the FGFR4 expression between tumor and normal tissues in pan‐cancer cohorts (TCGA + GTEx). ACC, adrenocortical carcinoma; BLCA, bladder urothelial carcinoma; BRCA, breast invasive carcinoma; CESC, cervical squamous cell carcinoma and endocervical adenocarcinoma; CHOL, cholangiocarcinoma; COAD, colon adenocarcinoma; DLBC, lymphoid neoplasm diffuse large B–cell lymphoma; ESCA, esophageal carcinoma; GBM, glioblastoma multiforme; HNSC, head and neck squamous cell carcinoma; KICH, kidney chromophobe; KIRC, kidney renal clear cell carcinoma; KIRP, kidney renal papillary cell carcinoma; LGG, brain lower grade glioma; LIHC, liver hepatocellular carcinoma; LUAD, lung adenocarcinoma; LUSC, lung squamous cell carcinoma; OV, ovarian serous cystadenocarcinoma; PAAD, pancreatic adenocarcinoma; PCPG, pheochromocytoma and paraganglioma; PRAD, prostate adenocarcinoma; READ, rectum adenocarcinoma; SKCM, skin cutaneous melanoma; STAD, stomach adenocarcinoma; TGCT, testicular germ cell tumors; THCA, thyroid carcinoma; UCEC, uterine corpus endometrial carcinoma; UCS, uterine carcinosarcoma. *Note:* **, *P* < 0.05; ***, *P* < 0.001.

To investigate the expression pattern of FGFR4 in breast cancer, we downloaded the TCGA‐BRCA cohort and found that FGFR4 expressed higher in breast cancer samples than in normal tissues (Figure 1A). Moreover, the high expression of FGFR4 was correlated with bigger tumor size, more lymph node metastasis, and advanced tumor stage, which were clinical parameters related to worse prognosis (Figure 1A). On the other hand, we explored the effect of FGFR4 level on breast cancer outcome and found that high FGFR4 level was correlated with shortened disease‐specific survival, relapse‐free survival, and progress‐free survival of breast cancer (Figure 1B). Besides, we further confirmed the prognostic value of FGFR4 in breast cancer with the pan‐BRCA cohorts from bc‐GenExMiner database. And the results showed that high FGFR4 level was correlated with shortened overall survival, distant metastasis‐free survival, and disease‐free survival of breast cancer (Figure 1C). Finally, we explored the FGFR4 expression differences between tumor and normal tissues in pan‐cancer cohorts (TCGA + GTEx) and found that FGFR4 was widely upregulated in multiple malignancies (Figure 1D). Taken together, the above findings indicated that FGFR4 is expressed highly in breast cancer and is associated with poorer outcome.

### 3.2 FGFR4 Inhibition Suppresses TNBC Progression

**FIGURE 2:**
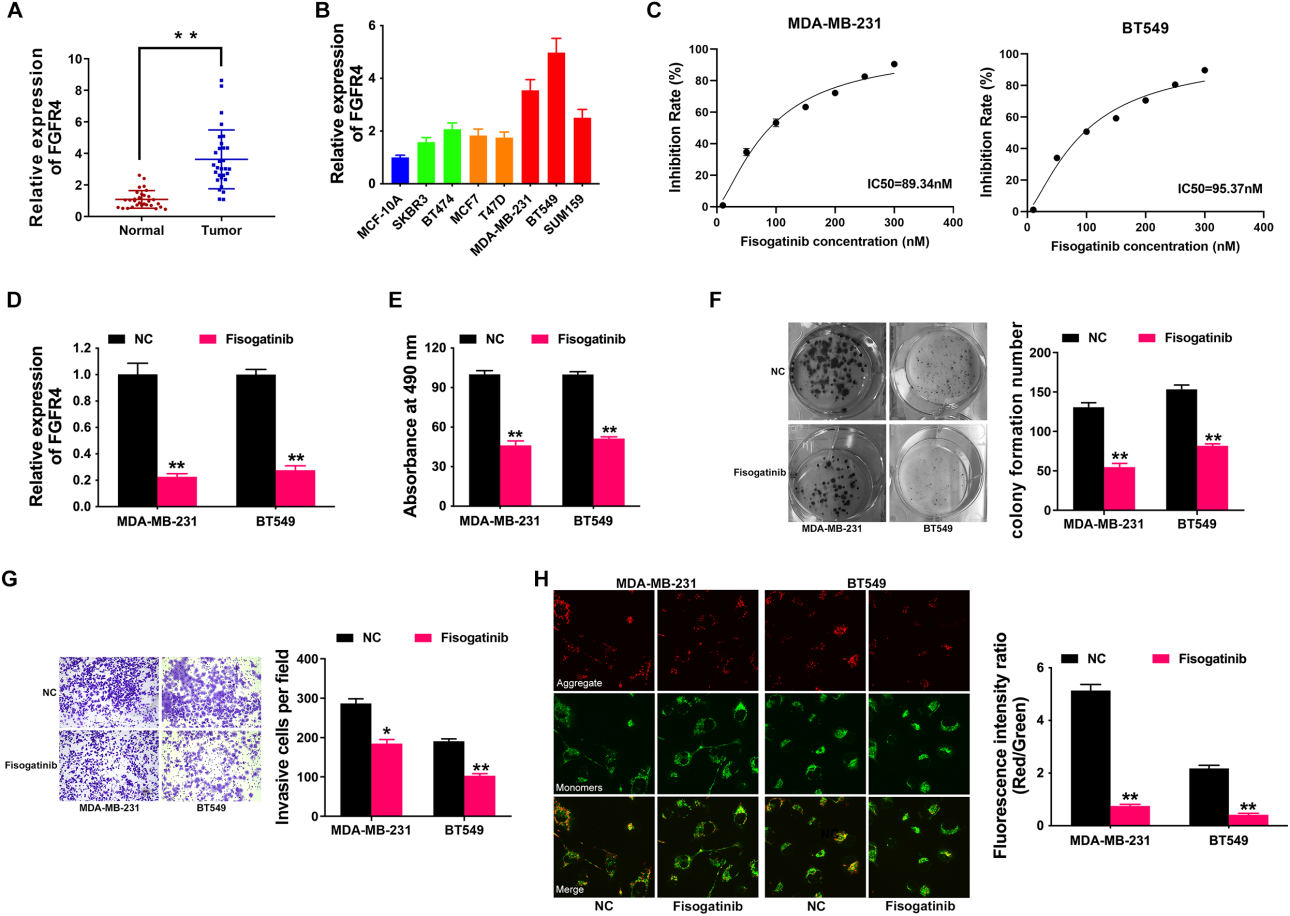
FGFR4 inhibition suppresses TNBC progression. (A) FGFR4 levels in 30 paired TNBC samples and normal adjacent samples by qRT‐PCR (Red: Normal; Blue: Tumor). (B) FGFR4 levels in breast cancer cells were detected (Blue: Normal breast cell line; Green: Her‐2 positive breast cancer cell lines; Orange: Luminal A/B breast cancer cell lines; Red: TNBC cell lines). (C) IC50 curve of TNBC cells treated with increasing doses of Fisogatinib for 24 h. (D) FGFR4 inhibition in TNBC cells was confirmed by qRT‐PCR. NC: Normal control, which was normalized to 1. (E) CCK‐8 assay after FGFR4 inhibition. NC: Normal control, which was normalized to 100%. (F) Represent image of colony formation assay after FGFR4 inhibition (left). Colony formation number was quantified by ImageJ software (right). (G) Represent image of Transwell assay after FGFR4 inhibition (left). Invasive cell number was quantified (right). (H) Represent image of JC‐1 staining for MMP measurement after FGFR4 inhibition (left). MMP level was quantified (right). *p < 0.05, **p < 0.01.

To confirm FGFR4 level in breast cancer, we detected FGFR4 expression in 30 paired TNBC samples and adjacent normal samples. Figure 2A showed that FGFR4 was indeed upregulated in TNBC samples compared to normal samples. Moreover, Figure 2B presented that FGFR4 also expressed highly in breast cancer cells, particularly in TNBC cell lines. Thus, we applied FGFR4‐specific inhibitor Fisogatinib to block the FGFR4 signaling pathway in these cell lines in order to explore the role of FGFR4 in TNBC. As shown in Figure 2C, the IC50 of Fisogatinib for treatment in two TNBC cell lines was89.34 nM and 95.37 nM. Therefore, we used 100 nM Fisogatinib to perform the following experiments. qRT‐PCR in Figure 2D revealed that Fisogatinib could successfully knockdown the level of FGFR4 in TNBC cell lines. CCK‐8 assay revealed that Fisogatinib suppressed TNBC proliferation (Figure 2E). Besides, Fisogatinib suppressed TNBC cell colony formation ability (Figure 2F). Moreover, Fisogatinib suppressed TNBC cell invasion, presented by Transwell assay in Figure 2G. Mitochondria plays a vital role in cell survival. We used JC‐1 staining to detect the function of FGFR4 on TNBC cell MMP. The results revealed that Fisogatinib decreased the level of MMP in TNBC cells (Figure 2H). All these results demonstrated that FGFR4 was closely correlated with TNBC cell growth, invasion, and survival.

### 3.3 FGFR4 Inhibition Suppresses TNBC Progression Through Regulating Fatty Acid Metabolism

**FIGURE 3:**
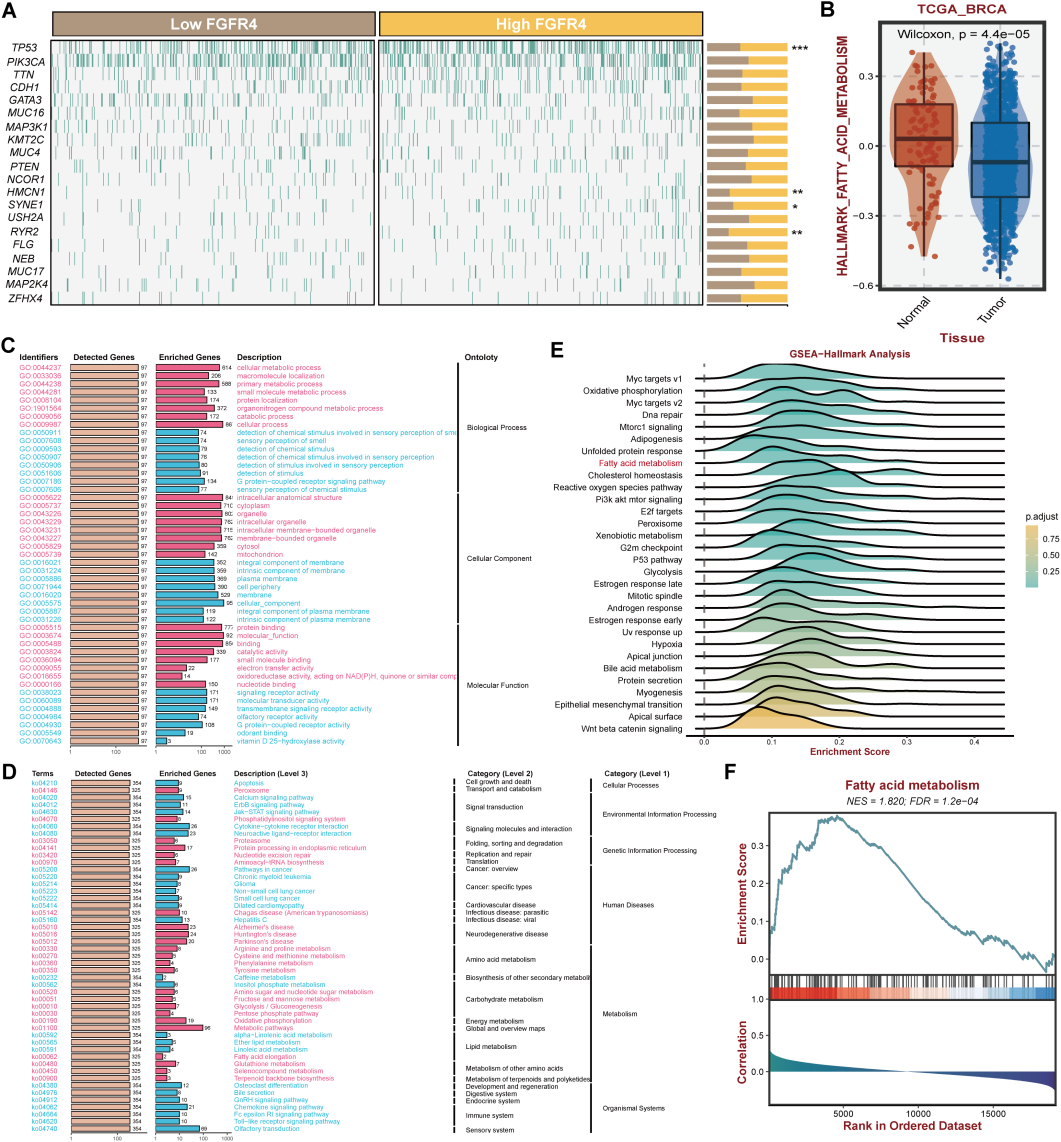
FGFR4 is associated with fatty acid metabolism in TNBC. (A) Mutation differences in the TCGA‐BRCA cohort based on the expression of FGFR4. (B) Boxplot of the activity score of hallmark fatty acid metabolism between tumor and normal tissues in the TCGA‐BRCA cohort. (C) GO enrichment analyses in the TCGA‐BRCA cohort based on the expression of FGFR4. (D) KEGG enrichment analyses in the TCGA‐BRCA cohort based on the expression of FGFR4. (E) GSEA analyses using 50 hallmark signatures in the TCGA‐BRCA cohort based on the expression of FGFR4. (F) The enrichment evaluation of the activity of fatty acid metabolism in breast cancer based on the expression of FGFR4.

Recently, fatty acid metabolism is getting more and more attention in cancer research. But the function and mechanism of FGFR4 in TNBC fatty acid metabolism are still unclear nowadays. Mutation differences in the TCGA‐BRCA cohort revealed that high expression of FGFR4 was related with more mutations in *TP53*, *HMCN1*, *SYNE1*, and *RYR2* (Figure 3A). Moreover, different activity score of hallmark fatty acid metabolism was discovered between tumor and normal tissues in the TCGA‐BRCA cohort (Figure 3B). Besides, both GO enrichment analyses (Figure 3C) and KEGG enrichment analyses (Figure 3D) in the TCGA‐BRCA cohort revealed that dysregulation of FGFR4 was correlated with cellular metabolic process, fatty acid metabolism included. GSEA analyses of the 50 hallmark signatures in the TCGA‐BRCA cohort also claimed the association between dysregulation of FGFR4 and fatty acid metabolism (Figure 3E). The GSEA enrichment evaluation based on the expression of FGFR4 showed that the fatty acid metabolism activity was closely related with breast cancer tumorigenesis (Figure 3F). All these results demonstrated that FGFR4 was closely correlated with fatty acid metabolism in breast cancer.

**FIGURE 4:**
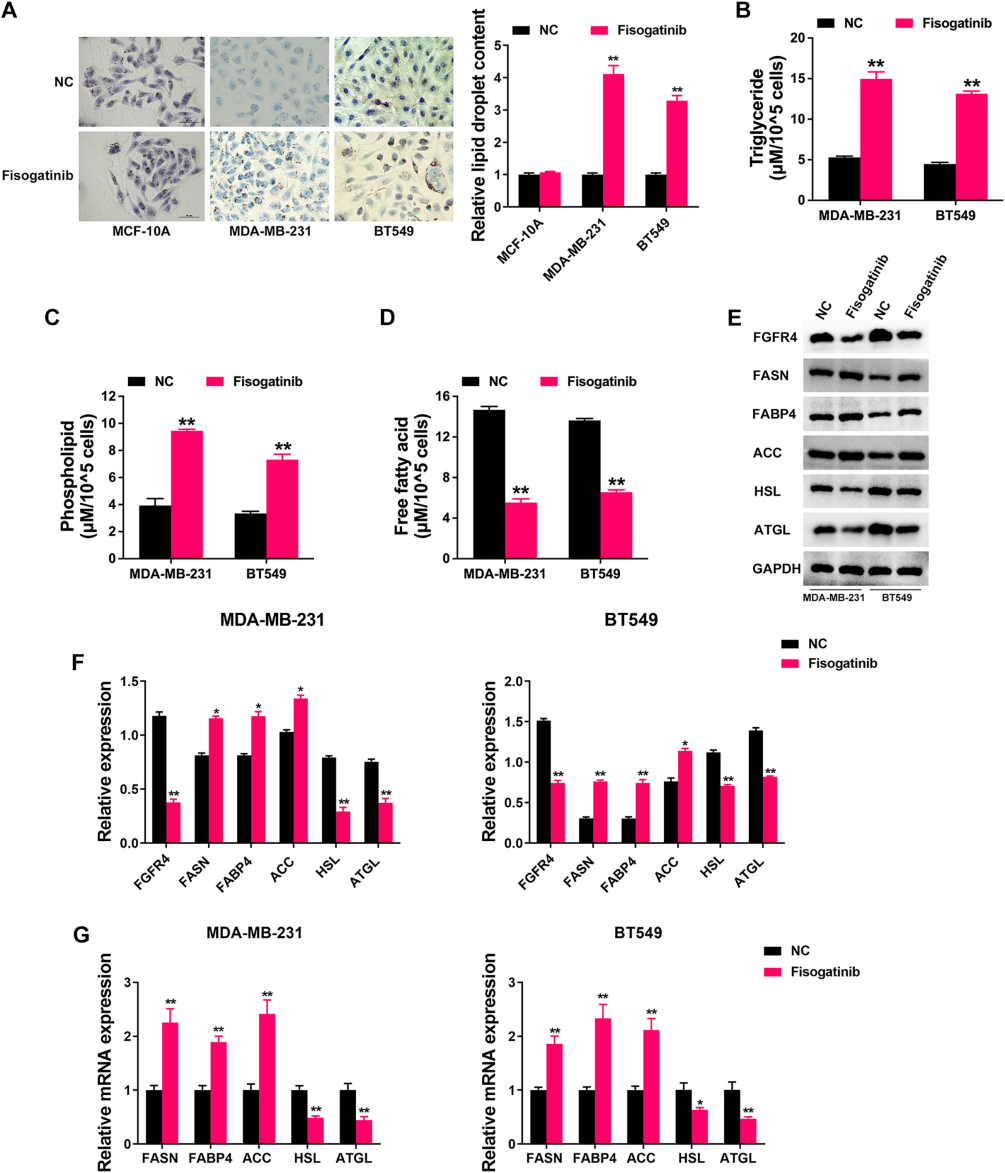
FGFR4 inhibition suppresses TNBC progression through regulating fatty acid metabolism. (A) Oil red O staining conducted to detect intracellular lipid contents after FGFR4 inhibition in cells (left). Intracellular lipid droplet contents were quantified by ImageJ software (right). (B) The intracellular TG levels were detected after FGFR4 inhibition. (C) The intracellular phospholipid levels were detected after FGFR4 inhibition. (D) The intracellular FFA levels were detected after FGFR4 inhibition. (E) Lipid metabolism‐related gene expressions were determined by Western blotting after FGFR4 inhibition in TNBC cell lines. (F) The Western blotting bands were quantified by ImageJ software. (G) The mRNA levels of lipid metabolism‐related genes were determined by qRT‐PCR after FGFR4 inhibition in TNBC cell lines. *p < 0.05, **p < 0.01.

Thus, we continued to find out the function of FGFR4 in TNBC fatty acid metabolism. Figure 4A showed that Fisogatinib didn't affect the lipid accumulation in normal breast cell, but increased lipid accumulation in TNBC cells, presented by Oil Red O staining. Moreover, Fisogatinib increased the intracellular levels of TG and phospholipid in TNBC cells, but decreased the levels of FFA (Figure 4B–D). Increased lipid biosynthesis or decreased lipolysis could cause elevation of intracellular lipid content. Therefore, we detected the expressions of key genes in fatty acid metabolism in order to investigate the underlying mechanism. We found Fisogatinib increased the expression of lipid biosynthesis‐related genes FASN, FABP4, and ACC, but reduced lipolysis‐related genes HSL and ATGL in TNBC (Figure 4E,F). qRT‐PCR also confirmed the effect of Fisogatinib on the mRNA levels of these genes in TNBC cell lines (Figure 4G). All these results demonstrated that FGFR4 played vital roles in TNBC fatty acid metabolism.

### 3.4 FGFR4 Regulates TNBC Progression Through the AKT/RYR2 Axis

**FIGURE 5:**
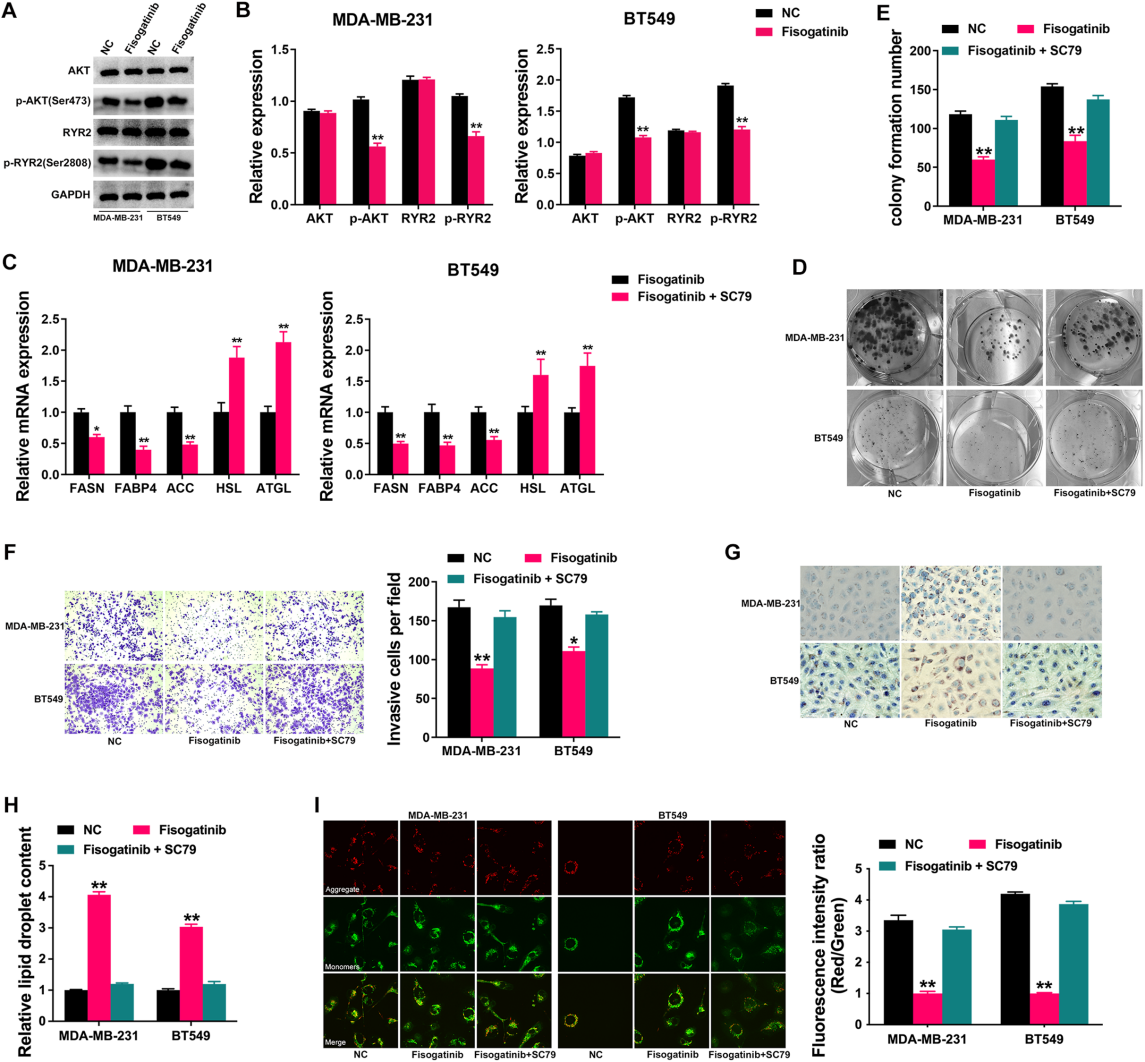
FGFR4 regulates TNBC progression through the AKT/RYR2 axis. (A) Western blotting was performed after FGFR4 inhibition in TNBC cell lines. (B) The Western blotting bands were quantified by ImageJ software. (C) The mRNA levels of lipid metabolism‐related genes were determined by qRT‐PCR after FGFR4 inhibition alone or combined with SC79 in TNBC cell lines. (D) Represent image of colony formation assay after treatment. (E) Colony formation number was quantified by ImageJ software. (F) Represent image of Transwell assay after treatment (left). Invasive cell number was quantified (right). (G) Represent image of Oil red O staining after treatment. (H) Intracellular lipid droplet contents were quantified by ImageJ software. (I) Represent image of JC‐1 staining for MMP measurement after treatment (left). MMP level was quantified (right). *p < 0.05, **p < 0.01.

Next, we continued to explore the mechanism of how FGFR4 regulated TNBC progression. We found that Fisogatinib decreased the AKT signaling activity, presented by reduced expression level of p‐AKT (Figure 5A,B). Besides, we found that Fisogatinib also downregulated the expression of p‐RYR2, a molecule predicted to be relevant to FGFR4 dysregulation as depicted in Figure 3A. Therefore, we used AKT activator SC79 to find out if FGFR4 realized its function through the AKT/RYR2 signaling. As shown in Figure 5C, treatment with SC79 reversed the effect of Fisogatinib on the levels of key genes of fatty acid metabolism. Colony formation assay revealed that Fisogatinib suppressed TNBC cell growth, which was rescued by SC79 (Figure 5D,E). Besides, Transwell assay showed that Fisogatinib suppressed TNBC cell invasion, which was also rescued by SC79 (Figure 5F). Oil Red O staining assay revealed Fisogatinib increased lipid accumulation of TNBC cells, which was reversed by SC79 (Figure 5G,H). MMP detection assay revealed that Fisogatinib decreased the level of MMP in TNBC cells, which was significantly reduced after being treated with SC79 (Figure 5I). The above results revealed that FGFR4 promoted TNBC progression partly through regulating fatty acid metabolism via the AKT/RYR2 axis.

## 4 Discussion

Increasing studies have implicated that FGFR4 is activated in most tumors and is involved in tumor growth, metastasis, and metabolism, acting as an oncogene [19, 20]. The overexpression of FGFR4 in breast cancer, which is instrumental in tumor progression, metastasis, and endocrine resistance, could potentially serve as a compelling target for therapeutic intervention [21, 22]. In breast cancer, FGFR4 was found to promote cell survival via activating the PI3K/AKT pathway [15]. Via sponging miR‐491‐5p, hsa_circRNA‐0001361 could up‐regulate FGFR4 expression level, leading to tumor progression in breast cancer [23]. Inhibition of FGFR4 could notably suppress tumor progression, suggesting that the strategic targeting of FGFR4 may serve as a potential approach in the treatment of breast cancer [24, 25, 26]. Here, we found that FGFR4 is expressed highly in breast cancer and is associated with poorer outcome (Figure 1). Moreover, inhibition of FGFR4 could suppress TNBC progression (Figure 2).

Metabolism reprogramming in tumor cells and tumor microenvironment supports the progression of tumors, which is a promising therapeutic target for cancer treatment [27, 28, 29, 30]. Deregulation of lipid metabolism is common in cancers, which is a hot topic in cancer research [31]. Recent studies have revealed that lipid metabolic reprogramming plays a vital role in TNBC tumorigenesis and development [32]. TNBC could promote carnitine palmitoyltransferase activity to enhance fatty acid oxidation (FAO) and ATP generation to increase energy homeostasis and cell viability, leading to tumor proliferation and metastasis [33]. TNBC cells could use fatty acids as an efficient energy source via FAO. For instance, Myc‐overexpressing TNBC shows upregulated FAO and ATP generation, leading to cell resistance against metabolic stress [34]. Moreover, activated FAO could promote energy homeostasis and cell viability, leading to TNBC metastasis [35]. In contrast, fatty acid synthesis (FAS) and lipogenic enzymes are found downregulated in TNBC [36, 37]. For example, TNBC reduces ACC activity to boost FAO flux, leading to enhanced EMT and metastasis 80. However, in some studies, blocking FASN and lipogenesis could have antitumor effects on TNBC [38, 39], indicating an intricate role of fatty acid metabolism in TNBC. For instance, proton pump inhibitor Omeprazole could selectively suppress FASN activity to induce TNBC cell apoptosis [40]. Therefore, investigating the mechanism of fatty acid metabolic remodeling could help design new biomarkers and metabolically targeted strategies for TNBC treatment.

FGFR4 has been reported to participate in breast cancer metabolism. Via activation of the FRS2‐ERK signaling, FGFR4 induced breast cancer doxorubicin (ADR) resistance and promoted glucose metabolism. Inhibition of FGFR4 restored the chemosensitivity of ADR‐resistant breast cancer cells and reduced the glycolytic flux [41]. In spite of the above findings, the functions and underlying mechanisms of FGFR4 in TNBC lipid metabolism remain incompletely discovered. Here, we found that FGFR4 was closely correlated with fatty acid metabolism in TNBC and that FGFR4 inhibition suppresses TNBC progression through regulating fatty acid metabolism (Figures 3 and 4).

FGF/FGFR signaling has been reported to promote tumor proliferation, invasion, and treatment resistance partly through inducing activation of the PI3K/AKT axis. Knockdown of FGFR4 could greatly inhibit tumor growth and metastasis via suppressing the PI3K/AKT signaling [42, 43]. Here, we found that FGFR4 regulates TNBC fatty acid metabolism and progression through the AKT/RyR2 axis and that FGFR4 inhibition decreased the AKT/RyR2 signaling activity (Figure 5).

RyR2 has also been recognized as a regulator of glucose metabolism [44, 45] and lipid metabolism [46, 47]. Researches have demonstrated that dysregulation of RYR2 is associated with development and progression of various cancers. RYR2 takes part in anti‐apoptosis pathway in a variety of malignancies. RyR2 has crucial functions in cell survival via regulating the calpain‐10‐mediated death pathway. Suppressing RyR2 significantly induced cell apoptosis [48]. In pancreatic cancer, inhibition of RYR2 suppressed cell proliferation, migration as well as invasiveness via induction of PTEN expression [49]. But few researches have explored the functions of RYR2 in TNBC so far. It is reported that PI3K/AKT signaling could induce the activation of RyR2 [50]. Here, we found that FGFR4 inhibition decreased the AKT/RyR2 signaling activity and eventually led to the reduction of RYR2 activity, indicating the regulation of FGFR4 on TNBC fatty acid metabolism and progression via the AKT/RYR2 axis (Figure 5).

## 5 Conclusion

This study claimed that FGFR4 acted as an oncogene in TNBC development and progression, which could act as a promising biomarker in TNBC diagnosis, prognosis, and treatment. And FGFR4 regulated TNBC tumorigenesis via regulating fatty acid metabolism through the AKT/RYR2 axis. Nowadays, there is still no drug‐targeted FGFR4 that has gained clinical approval. Further comprehensive research is warranted to facilitate the development of FGFR4‐targeted agents, which could potentially expedite the clinical application of FGFR4 for the treatment of malignancies.

## Author Contributions

**Jinhui Ye:** conceptualization (equal), data curation (equal), formal analysis (equal), writing – original draft (equal). **Song Wu:** conceptualization (equal), data curation (equal), writing – original draft (equal). **Qiang Quan:** data curation (equal), investigation (equal), visualization (equal). **Feng Ye:** formal analysis (equal), investigation (equal). **Jinhui Zhang:** formal analysis (equal), validation (equal). **Cailu Song:** validation (equal). **Yidan Fan:** investigation (equal). **Huijiao Cao:** project administration (equal), writing – review and editing (equal). **Hailin Tang:** project administration (equal), writing – review and editing (equal). **Jianfu Zhao:** conceptualization (equal), project administration (equal), writing – review and editing (equal).

## Ethics Statement

This study was approved by The Ethics Committee of the Sun Yat‐Sen University Cancer Center, and 1964 Helsinki Declaration was followed.

## Consent

Patients involved had written informed consent forms.

## Conflicts of Interest

The authors declare no conflicts of interest.

## Data Availability

All data and materials used in this study are available from the corresponding author upon reasonable request.
